# Realizing the full reservoir operation potential during the 2020 Yangtze river floods

**DOI:** 10.1038/s41598-022-06801-8

**Published:** 2022-02-18

**Authors:** Hairong Zhang, Yanhong Dou, Lei Ye, Chi Zhang, Huaming Yao, Zhengfeng Bao, Zhengyang Tang, Yongqiang Wang, Yukai Huang, Shuang Zhu, Mengfei Xie, Jiang Wu, Chao Shi, Yufeng Ren, Dongjie Zhang, Biqiong Wu, Yufan Chen

**Affiliations:** 1 China Yangtze Power Co., Ltd., Yichang, 443133 China; 2 Hubei Key Laboratory of Intelligent Yangtze and Hydroelectric Science, Yichang, 443133 China; 3 School of Hydraulic Engineering, Dalian University of Technology, Dalian, 116024 China; 4 Changjiang River Scientiffic Research Institute, Wuhan, 430074 China; 5 School of Geography and Information Engineering, China University of Geosciences, Wuhan, 430074 China; 6 Kunming Power Exchange Center Co., Ltd., Kunming, 650011 China; 7 Yunnan Power Grid Co., Ltd., Kunming, 650011 China

**Keywords:** Hydrology, Natural hazards

## Abstract

Five severe floods occurred in the Yangtze River Basin, China, between July and August 2020, and the Three Gorges Reservoir (TGR) located in the middle Yangtze River experienced the highest inflow since construction. The world’s largest cascade-reservoir group, which counts for 22 cascade reservoirs in the upper Yangtze River, cooperated in real time to control floods. The cooperation prevented evacuation of 600,000 people and extensive inundations of farmlands and aquacultural areas. In addition, no water spillage occurred during the flood control period, resulting in a world-record annual output of the TGR hydropower station. This work describes decision making challenges in the cooperation of super large reservoir groups based on a case-study, controlling the 4th and 5th floods (from Aug-14 to Aug-22), the efforts of technicians, multi-departments, and the state, and reflects on these. To realize the full potential of reservoir operation for the Yangtze River Basin and other basins with large reservoir groups globally, we suggest: (i) improve flood forecast accuracy with a long leading time; (ii) strengthen and further develop ongoing research on reservoir group cooperation; and (iii) improve and implement institutional mechanisms for coordinated operation of large reservoir groups.

## Introduction

Floods are the most frequent natural disaster globally affecting countless lives and property^1^. Reservoirs, one of the most efficient infrastructures in flood control^2^, were built extensively, such that, cascade-reservoir groups are common in large basins, such as the Nile^3^, the Yangtze River (alias: Changjiang)^4^, the Yellow River^5^, etc. When encountering a severe flood, cascade-order impounding of floods for cascade-reservoir groups can reduce the flood control pressure of a single reservoir and improve the flood control ability of the whole basin^6^. However, each reservoir must manage a balance between flood risk and economic benefit such as power generation and shipping, and benefits gained from different reservoirs vary between each other^7,8^. According to the World Commission on Dams^9^, most large reservoirs worldwide cannot produce benefits the authorities are satisfied with. As a result, the cooperation of cascade-reservoir groups has been extensively considered by hydrologists and decision makers. However, the connection between hydrologists and decision makers is relatively weak, although there are many studies on reservoir cooperation^10–13^, little feedback and reflection has emerged from users. In this work, we describe challenges, achievements and reflections of the cooperation practice of the world’s largest cascade-reservoir group in the upper Yangtze River when facing a flood larger than that with a 100-year recurrence interval.

**Figure 1:**
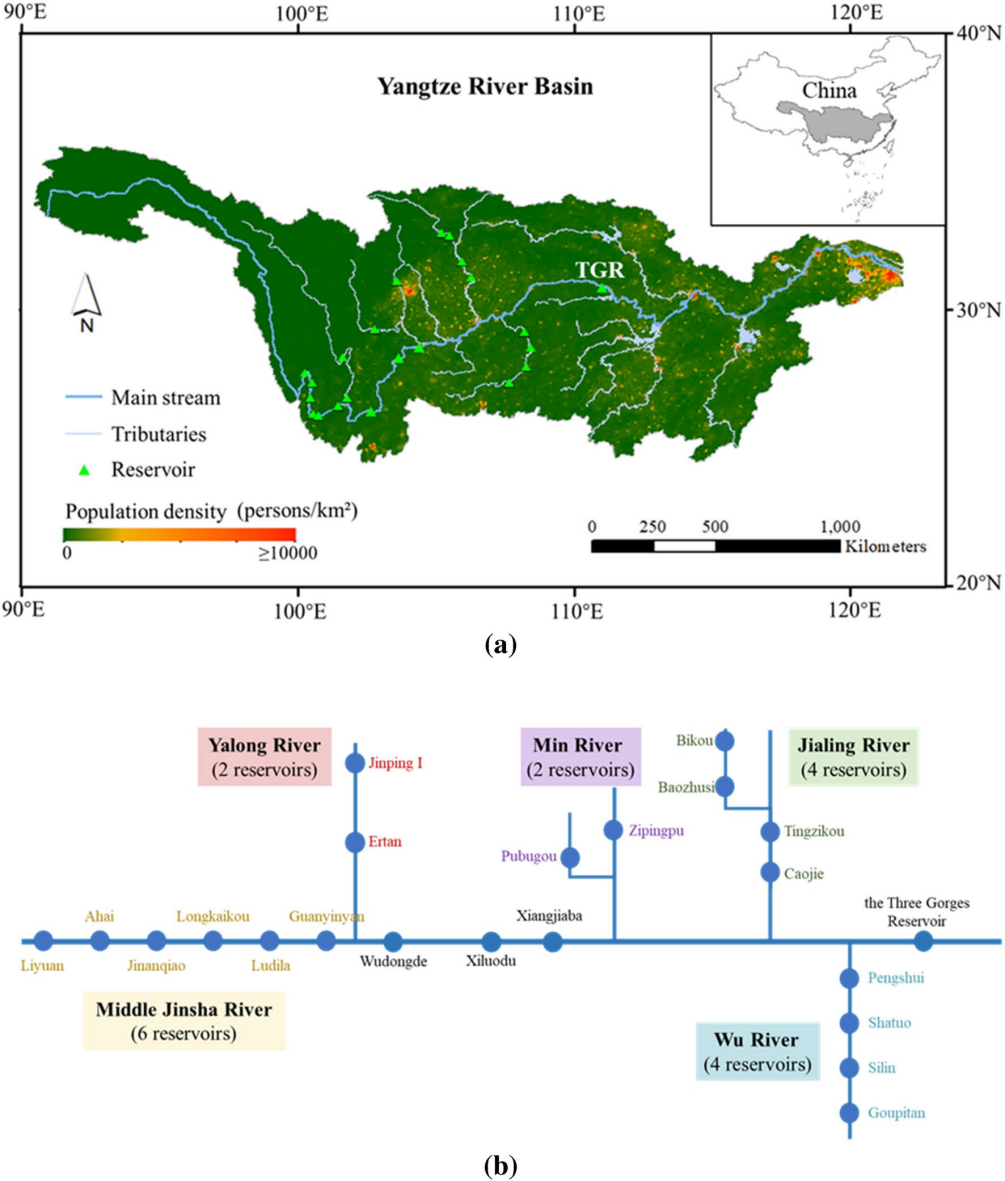
(**a**) The geographical location, streams, reservoirs and population density of Yangzte River Basin generated by ArcGIS 10.2. (**b**) Sketch map of 22 reservoirs on the upper Yangtze River included in the joint operation until 2020, where the circles represent reservoirs.

The Yangtze River, the third-longest river in the world, plays an paramount role in the economy, energy, and ecology of China^14–17^; it has a basin area of 1.8 × 10^6^ km^2^ that serves a population of nearly 50 million people, generates 40% of the China's Gross Domestic Product (GDP), and outputs 30% of China's grain. The Yangtze River Basin is shown in Fig. 1a, where the distribution of population density is developed by Socioeconomic Data and Applications Center^18^. Reservoir groups on the upper Yangtze River are vital for flood control in the middle and lower regions^19,20^. The largest cooperation system of reservoirs in the world is constructed in the upper Yangtze River Basin, as shown in Fig. 1b, which counts for 22 cascade reservoirs with a total flood control capacity of 38.7 × 10^9^ m^3^^21^. The system takes the Three Gorges Reservoir (TGR; largest installed capacity in the world) as the core reservoir, the cascade reservoirs in the lower Jinsha River, including Wudongde (7th), Xiluodu (4th), and Xiangjiaba (11th), as the main reservoirs, and the cascade reservoir groups in the middle Jinsha River (six reservoirs), Yalong River (two reservoirs), Min River (two reservoirs), Jialing River (four reservoirs), and Wu River (four reservoirs) as the coordinating reservoirs.

## Challenges

### Severe floods of Yangtze river

Rainfall distribution is grossly inhomogeneous in both spatial and temporal aspects in the Yangtze River Basin, benefiting from its location in the subtropical monsoon climate zone on the east coast of Eurasia^22,23^. As a result, floods occur frequently in the Yangtze River Basin and are usually characterized by a strong sudden occurrence, significant areal extent, and massive loss of lives and property^24,25^.

**Figure 2:**
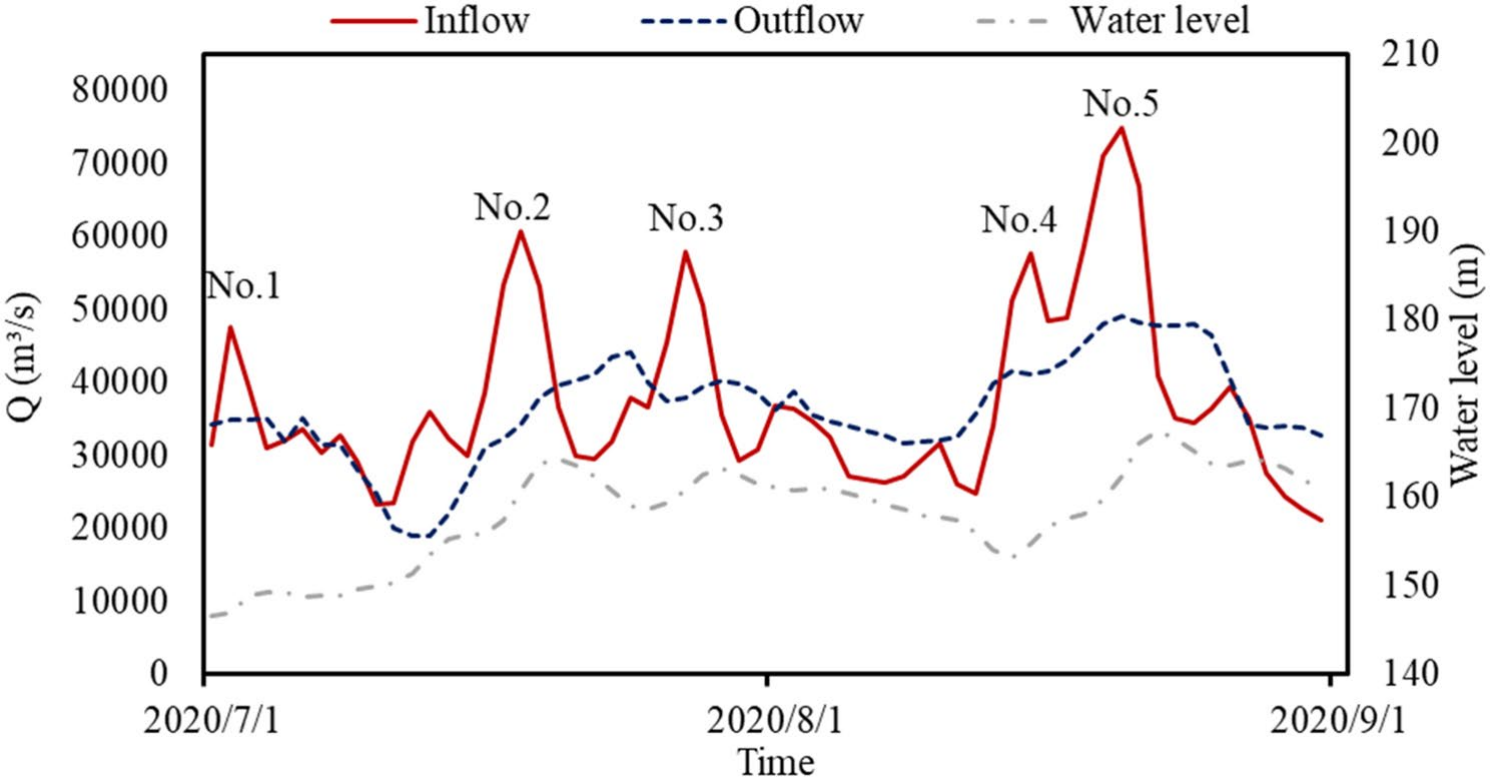
Outflow, inflow, and water level of TGR during 2020 flood season.

In 2020, five severe floods occurred between July and August along the Yangtze River as shown in Fig. 2. The TGR, located in the middle Yangtze River, experienced the highest inflow of 74,600 m^3^/s since its construction. Among the five numbered floods, 4th and 5th floods (from Aug-14 to Aug-22) were caused by two continuous rainfalls characterized by long duration, high intensity, and overlapping rainfall fields which covered most of the upper Yangtze River. During the rainstorm, the 5-day accumulated rainfall exceeded 400 mm, meaning that half of the average annual rainfall fell in just five days. As a result, flood peaks of the upper Yangtze River tributaries would encounter before entering the TGR and a flood larger than that with a 100-year recurrence interval would be formed without reservoir operation.

### Complex operating decision of super large reservoir groups

#### Cooperation of reservoirs in the mainstream and tributaries

According to the joint operation scheme, when a severe flood occurs, the TGR and the 21 upstream reservoirs will coordinate with each other to control the flood. This means that the upstream reservoirs would not only perform their own flood control tasks, but also reserve part of their flood control capacity, known as reserved flood control capacity, to cooperate with the TGR to mitigate flooding in the middle and lower regions of the Yangtze River. The mainstream flood may be comprised of two or even more floods in the tributaries owing to the numerous tributaries and their frequent flood encounters in the Yangtze River^26^. Considering the complicated composition of mainstream floods, various cooperation schemes should be used for the reservoirs on the mainstream and tributaries. Additionally, the distance between each reservoir group is significant as shown in Fig. 1a, which increases the inherent uncertainty of flood routing and rainstorm displacement direction forecasting. Therefore, the real-time joint operation of the super large reservoir groups faces the problem of how to determine the utilization order of the reserved flood control capacity, as well as how much to use and maintain.

#### Impact on stable power supply

The cascade hydropower stations on the upper Yangtze River are the key power sources of China^27^. The theoretical and exploitable hydropower resources in the Yangtze River Basin are 2.68 × 10^9^ kW and 2.35 × 10^9^ kW, respectively, out of which the upper Yangtze River accounts for more than 90%. Considering the Xiluodu-Xiangjiaba-Three Gorges cascade stations as an example, these three stations are responsible for power supply to nine of the most economically developed provinces and cities in China, including Guangdong, Jiangsu, Zhejiang, and Shanghai^28^. However, when cooperating with the downstream or mainstream reservoirs, the upstream or tributary reservoirs must reduce discharge to release the reserved flood control capacity, leading to a decrease in the output of the hydropower stations and making it difficult to generate stable power as specified in the original generation schedule^29–31^. With respect to the status of the cascade hydropower stations on the upper Yangtze River, erratic hydropower supply affects other power sources in the power grid like dominoes, particularly by decreasing the output during the peak summer months. Therefore, the second problem that the real-time joint operation of super large reservoir groups faces is how to adjust the generation schedule.

#### Impact on safe operation

During a severe flood, the increasing inflow and flexible discharge of reservoirs quickly change the water levels of both upstream and downstream dams, impacting the safe operation of water conservation projects^32^ and shipping transportation. Taking Xiangjiaba reservoir as an example, if the daily variation in the water level exceeds 4 m, it leads to reservoir bank instability^33^, and if the water level variation is too large in the downstream dam, shipping safety is affected^8^. Therefore, precise control of water level variation is necessary, particularly at night, adding additional uncertainties owing to fewer staff.

#### Coordination of multiple departments

**Figure 3:**
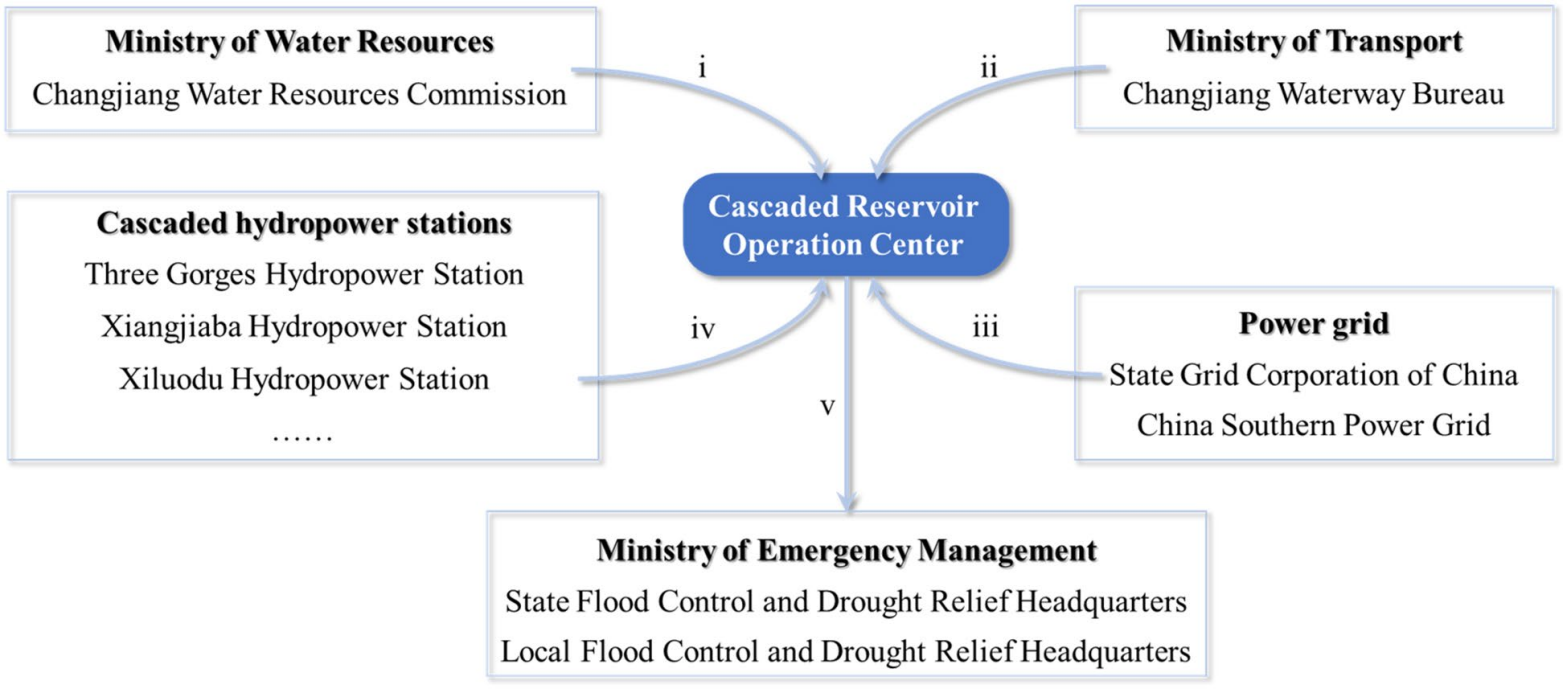
The relationship between six types of water-related departments, where i to iv indicate the objectives and/or requirements of each corresponding type of department for reservoir cooperation, and v indicates the submission of the cooperation scheme ensemble from Cascaded Reservoir Operation Center (CROC) to Flood Control and Drought Relief Headquarters (FCDRH) considering the above objectives and requirements. The final cooperation scheme is determined through multi-department consultation under the leadership of FCDRH.

Each reservoir included in the upper Yangtze River cooperation system is responsible for multiple simultaneous missions, such as flood control, power generation, and shipping^7,8^, etc. The operation of reservoirs affects the competition-relation benefits of multiple departments^34^. For example, when facing a flood, the objective of Changjiang Water Resources Commission (CWRC) of the Ministry of Water Resources (i in the Fig. 3) is mitigating the threat of flood to the protection objectives in the basin, such as populated localities, factories and farmlands. For this purpose, impounding and/or discharging of reservoirs will cause frequent changes of water level and flow, which affects shipping and power generation significantly. Both the Changjiang Waterway Bureau (CWB) of the Ministry of Transport and power grid corporations hope to reduce the duration of such abnormal operation as much as possible (ii and iii in the Fig. 3). Additionally, it is necessary to consider maintenance schedules and upper limits of transmission for each hydropower station (iv in the Fig. 3). Therefore, one problem reservoir cooperation decision making faces, is how to manage and balance the objectives and requirements of multi-departments.

## Achievements of cascade-reservoir cooperation

### Flood control

**Figure 4:**
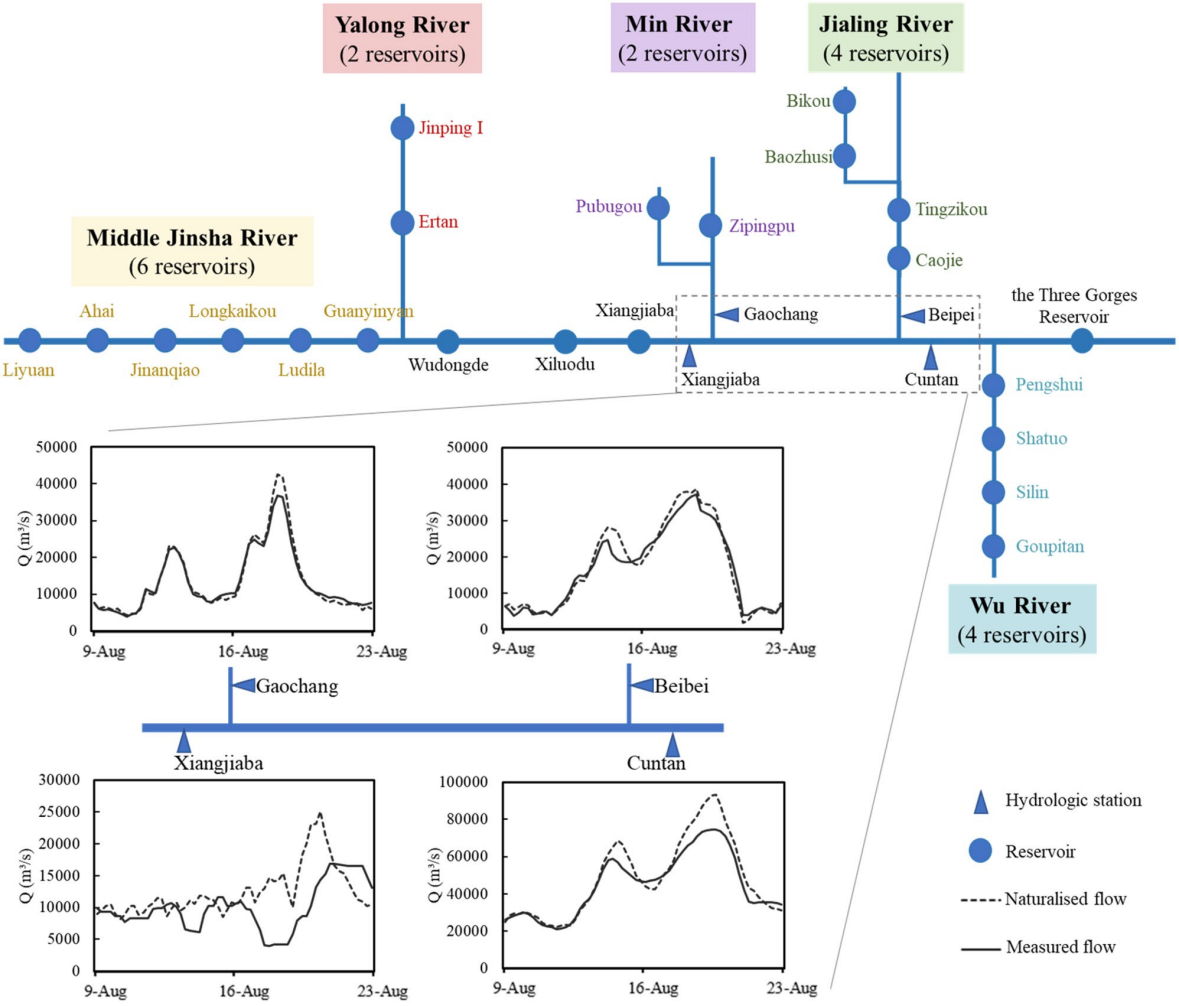
The triangles represent the hydrological stations; and naturalized and measured flows of major hydrological stations in the upper Yangtze River during 4th and 5th floods, where the naturalized flow of the stations on the Jinsha River (Xiangjiaba), Min River (Gaochang), Jialing River (Beibei), and upstream of the TGR (Cuntan) is obtained using the Intelligent Changjiang Decision Support System (ICDSS) according to the actual operational data of upstream reservoirs and considering the flood travel time and water balance.

To prevent the 1st, 2nd, and 3rd floods of the Yangtze River in 2020, 20.5 × 10^9^ m^3^ of the flood control capacity of the 22 joint-operation reservoirs was used, accounting for 53% of the total flood control capacity. On this basis, to prevent the 4th and 5th floods, the cascade reservoir groups cooperated on a large scale again and held up 19.0 × 10^9^ m^3^ of flood volume. There is 8.2 × 10^9^ m^3^ of flood volume held up by the 21 reservoirs upstream of the TGR, as shown in Fig. 4, which prevented flood peak encounters of the Jinsha River (Xiangjiaba Station), Minjiang River (Gaochang Station), and Jialing River (Beibei Station). As a result, before the flood enters the TGR (Cuntan Station), the flood peak with a 90-year recurrence interval (87,500 m^3^/s) was reduced to 20 years (74,600 m^3^/s), the flood volume with a 130-year recurrence interval was reduced to 40 years (36.00 × 10^9^ m^3^), and the peak river stage was reduced by 3 m. In this manner, flood control pressure at the tail of the TGR was significantly mitigated. The other 10.8 × 10^9^ m^3^ of flood volume was held up by the TGR, which reduced the flood with a 50-year recurrence interval (40.00 × 10^9^ m^3^) to an ordinary flood and avoided the utilization of the Jingjiang Flood Diversion Area. The above flood control cooperation prevented significant direct property loss in the middle Yangtze River, such as the evacuation of 600,000 people and inundation of 330 km^3^ of farmlands and more than 70 km^3^ of aquaculture areas.

### Hydropower generation

In response to flood control, power grid companies adjusted the hydropower generation schedules of relevant reservoirs efficiently and scientifically to ensure stable power supply, efficient use of water resources, and greater power generation. Taking the operation of the Xiangjiaba Reservoir during the 4th and 5th flood as an example, the Xiangjiaba Reservoir had to reduce the discharge and hold the flood in order to cooperate with the TGR, resulting in decreased flow through the turbine and the plummeting power generation. The State Grid quickly adjusted the power generation schedule, that is, permitted the output of the Xiangjiaba hydropower station to be reduced to 2.2 × 10^6^ kW at 17:00 on Aug. 17, and subsequently, the output was increased to the rated power at 9:00 a.m. on Aug. 19. During the entire process, no water spillage occurred. As a result, the Xiluodu-Xiangjiaba cascade hydropower stations and the single station of the TGR generated electricity outputs of 3.20 × 10^10^ kWh and 1.67 × 10^10^ kWh, respectively in August 2020, which are 49% and 42%, respectively more than the 10-year average in the same period. In addition, the TGR hydropower station generated electricity output of 1.12 × 10^11^ kWh in 2020, hitting a world record for annual output of a single hydropower station^35^.

### Safe operation

The water level variations of both the upstream and downstream dams met the corresponding regulation during the flood control cooperation owing to the benefit derived from the forecast of precipitation and hydrology and the simulation of hydrodynamics and reservoir operation. The daily water level variation of the Xiluodu and Xiangjiaba reservoirs were 4.95 m (5 m in regulation) and 3.73 m (4 m in regulation) respectively, which is the largest since their construction. From July to September 2020, more than 4500 ships and 15 million tons of cargo successfully passed through the waterway in an orderly manner.

## Reflections

Real-time cooperation was adopted for cascade reservoir groups for controlling floods in 2020, realizing the full reservoir operation potential. During the real-time cooperation, conventional operation schedules and regulations are always not applicable. Below are three key reflections from the reservoir operation during the 2020 Yangtze River flood.

### Accurate forecast of precipitation and flow

Technicians from the CROC of China Yangtze Power Co., Ltd. forecasted the extreme precipitation and floods two weeks ahead of the 4th flood qualitatively, 2 floods with both peaks of more than 60,000 m^3^/s a week ahead qualitatively, and the discharge and appearance times of the flood peaks 3–5 days ahead quantitatively, using the system that is responsible for precipitation and hydrology forecasting in ICJDSS (details can be seen in “Methods and materials” section). Owing to the lower accuracy of the forecast with a longer leading time^36^, the forecast changed from qualitative estimation to quantitative prediction with the gradual approach of the forecast target. The quantitative forecast performance is presented in Table 1.

**Table 1:** Quantitative forecast performance during 4th and 5th floods in 2020.

No	Measured		Forecasted			Evaluation	
	Peak flow (m^3^/s)	Peak time	Leading time	Peak flow (m^3^/s)	Peak time	Error of peak flow	Error of peak time
4	62,000	Aug-15	5 day	50,000	Aug-16	− 19.35%	1 day
			1 day	62,000	Aug-15	0	0
5	74,600	Aug-20	5 day	62,000	Aug-20	− 17.33%	0
			1 day	74,600	Aug-20	0	0

Following the qualitative precipitation forecast of the next 7–14 days, the water level of the TGR dropped from 163.36 m on Jul-29 to 153.03 m on Aug-14 (its flood limited water level is 145 m) by pre-discharging; further, 17.75 × 10^9^ m^3^ of the flood control capacity was reserved, accounting for 80% of the total flood control capacity of the TGR, which ensured adequate preparation for flood control and time to adjust the power generation schedule. Following the quantitative flood forecast of the next 3–5 days, the reserved flood control capacity of upstream reservoirs (mentioned in section “Complex operating decision of super large reservoir groups”) was released to hold up a portion of the flood and stagger flood peaks through real-time joint operation.

### Scientific cooperation of cascade reservoir groups

In recent years, research efforts on the joint operation and coordinated management of reservoirs in the upstream Yangtze River have continued to increase, and the application of scientific cooperation of cascade reservoir groups has also been enriched.

In terms of a cooperation scheme, the flood control capacity of the reservoirs in the upper Yangtze River is reserved for cooperation with other reservoirs, and the reserved capacity is gradually released based on the flood season stages^37,38^ and the reservoir classifications. Specifically, (1) In the main flood season, the necessary capacity is reserved to hold the possible floods, and gradually released when the inflow shows a declining trend during the post-flood season. (2) The release order of each reservoir depends on flood encounter situations, its own flood control tasks, and its role in cooperation with the TGR for flood control of the middle and lower Yangtze River regions.

In terms of real-time cooperation, existing technology is used to predict or identify the flood type^39^, whereas the flood control capacity of cascade reservoirs is determined by regulating each type of flood in the entire basin during the design stage. Therefore, in the case where there is no need to prevent various flood types simultaneously, some reservoirs have surplus flood control capacity. The surplus capacity of upstream reservoirs share the flood control tasks of the downstream reservoirs, and as a result, the water levels of the downstream reservoirs can be raised to increase the benefit without increasing the flood risk of the entire basin^40^. In this manner, reservoirs can be compensated by the capacity of each reservoir based on the relatively accurate forecast and the joint operation pattern, so that the water levels of some reservoirs with significant benefits in the flood season can be relatively high and flexibly controlled without strictly following a single flood limit water level. For instance, with the most significant benefits in the Yangtze River, the legislative flood limit water level of TGR ranges from 145.0 to 146.5 m. Between the 3rd and the 4th flood, the water level of the TGR was reduced to 153.03 m by maximum discharge through turbines, rather than the flood limit water level of 145 m with water spillage, as the upstream reservoirs could hold up part of the flood^41,42^. In Fig. 4, it can be observed from the difference between the naturalized and measured flows at the Xiangjiaba Station that the Xiangjiaba reservoir and its upstream reservoirs play an important role in flood storage and flood peak staggering; at the Gaochang and Beibei Stations, the reservoirs in the Min and Jialing Rivers also hold up part of the flood, so that the flood peaks of the Gaochang and Beibei Stations with recurrence intervals of 40–50 years (40,600 m^3^/s) and 10–15 years (38,400 m^3^/s), respectively were reduced to 10–15 years (37,500 m^3^/s) and 10 years (37,400 m^3^/s), respectively.

### Regulations for multi-department coordination

The Yangtze River reservoirs can be flexibly used to realize the potential benefits safely by joint operation, which is inseparable from the state macro-coordination (state FCDRH) and the cooperation of the local governments (local FCDRH), the basin management organization (CWRC), the transport department (CWB), hydropower stations and power grid companies. As an intermediary of multiple departments, CROC is responsible for the technical support of generating an ensemble of potential cooperation schemes and information transport and management. During the 4th and 5th flood, the above departments held 10 consultations on matters including real-time hydro-meteorological monitoring and forecast information, real-time joint operation of reservoirs, and temporary adjustment of the hydropower generation schedule. Reservoirs discharged flexibly in real-time, rather than strictly following rigid operating schedules and regulations.

To ensure a scientific, unified, and coordinated operation of the cascade reservoir groups in the Yangtze River Basin, a series of relevant regulations have been established in recent years. In the aspect of monitoring and management, the institutional mechanism dedicated to information sharing, benefits compensation^43^, and risk control^44,45^ for the reservoir groups has been established. In terms of the administrative system, the coordination mechanism dedicated to multiple departments involving water resources, power, shipping, and environmental protection has been improved^46^ based on the existing flood control organization^47^. On the basis of continuous improvement of the above mechanisms and systems, the legislation of the Law of the People's Republic of China on the Protection of the Yangtze River was promoted, which was promulgated on December 26, 2020, and came into force on March 1, 2021^48^.

## Call for action

Largely drawing on the reflections reported based on the reservoir cooperation during the 2020 Yangtze River, we suggest the following action points to realize the full potential of the reservoir operation of the Yangtze River basin and other basins with large reservoir groups in the world:

1. Improve flood forecast accuracy with a long lead time. Accurate flood forecast is the premise of joint and precise operation of reservoir groups, adjustment of the power generation schedule, and multi-department cooperation.
2. Continue to study the cooperation of reservoir groups. As the number of reservoirs included in the joint operation increases constantly, new situations and challenges with new requirements will gradually become prominent. It is necessary to conduct research on the cooperation of reservoir groups and to formulate scientific counter measures.
3. Improve and implement regulations for the coordinated operation of large reservoir groups. A powerful coordination regulation has the potential to guarantee effective implementation of the scientific operation of super large reservoir groups.

## Methods and materials

Decision making of reservoir cooperation in upper Yangtze River Basin includes three steps. Firstly, the preliminary ensemble of cooperation schemes is obtained by Cascaded Reservoir Operation Center (CROC) through Intelligent Changjiang Decision Support System (ICDSS), powered by China Yangtze Power Co., Ltd. Secondly, departments through rounds of consultation to determine the final cooperation scheme. Finally, ICDSS reanalysis will be used to summarize the experience after the flood.

### Intelligent Changjiang decision support system

Intelligent Changjiang Decision Support System is vital for the reservoir groups to optimally achieve the comprehensive operation objectives of flood control, power generation, shipping and ecology by building a tailored technology framework. ICDSS includes four main components: a data acquisition and management system; a set of interlinked models and data-model-user interactive interface; a socioeconomic evaluation system; and crisis management plans.

#### Data acquisition and management system

This system is responsible for automatic collecting and managing unstructured data (e.g., hydro-meteorology data, operation data and grid load data) and semi-structured data (e.g., operation regulations and instructions). In the upper Yangtze River Basin, there are 1005 hydrological stations (of which 372 are managed by local Hydrological Bureau and 633 by CRCC), 12,129 precipitation stations (of which 730 are national stations, 11,000 are local stations and 399 are managed by CROC), and 37 national ground-based radars. The failure rate of the above stations was 1.7% in the past three years.

#### Interlinked models and data-model-user interactive interface

- Simulation of upstream reservoir operation: The operation of cascade hydropower stations in the upper Yangtze River changes the propagation characteristics of natural flow, as a result, the accuracy of hydrological forecasting is affected. Therefore, this system is responsible for mining rules of impounding and discharging for upper reservoirs that is not included in joint operation and anticipating discharge of these reservoirs.
- Hydro-meteorological forecast and discharge routing simulation^49–52^: The system is composed of the Xin’anjiang model with 377 subunits, the one-dimensional hydrodynamic model, and the error correction module. Each module of the system runs automatically but can also be supplemented by human–machine interaction functions depending on the experience of the forecasters.
- Optimal cooperation of cascade-reservoir groups with different leading time^53–56^: This system is responsible for generating the optimal ensemble of reservoir cooperation based on hydro-meteorological forecast with different leading time. The NSGA-II is used to obtain the optimal cooperation schemes and Pareto solutions of corresponding objectives. Hedging theory and marginal benefit theory are used to analyze the competition between risk and benefit. As the update of forecast information with different leading time, the ensemble of reservoir cooperation is rolling executed.

#### Socioeconomic evaluation system

This system is responsible for evaluation and reanalysis the cooperation process of cascade-reservoir groups. By analyzing the contribution of each reservoir in cascade-reservoir groups to flood control, power generation, shipping and ecology, the influence of man-made and natural factors in the comprehensive achievements is clarified, and the operation scheme will be revised and improved based the re-evaluation of results.

#### Crisis management plans

There are 22 crisis management plans for failures of automatic control systems, equipment and power, as well as kinds of accidents and emergencies.
